# The script concordance test: an adequate tool to assess clinical reasoning?

**DOI:** 10.1007/s40037-018-0437-6

**Published:** 2018-06-12

**Authors:** Eugène J. F. M. Custers

**Affiliations:** Centre for Research and Development of Education, UMC Utrecht School of Medical Sciences, Utrecht, The Netherlands

In this issue of *Perspectives on Medical Education*, Lubarsky et al. investigate the possibility that test takers can obtain artificially inflated test scores on the Script Concordance Test (SCT) by strategically selecting options, rather than by using their knowledge to arrive at the correct answer [1]. The Script Concordance Test is a test to assess clinical reasoning, consisting of items that require students to judge the change in likelihood of a diagnostic hypothesis (provided to them) given a new piece of information (also provided to them) on a five-point Likert scale, ranging from −2 ([almost] rules out the hypothesis) through 0 (does not change its likelihood) to +2 (hypothesis now [almost] certain) [2]. For each item, the test takers’ response is compared with the composite judgment of a panel of experts, and credit points are assigned in accordance with the proportion of experts selecting the same response when test norms are constructed. If the testee selects the same response as the majority of experts, he/she obtains the maximum number of credit points. If the testee sides with a minority of experts, points are detracted. Otherwise, no points are awarded at all. By running a simulation, Lubarsky and colleagues [1] demonstrate that test takers who consistently choose the ‘0’ option (no change in likelihood) can artificially inflate their scores from −2.1 SD below the average of an actual sample of respondents—the score obtained by random guessing,—to −0.7 SD below this average. It may be argued that this form of ‘gaming the test’ by consistently selecting the ‘0’ option is contrived. In fact, it is an inevitable consequence of using a computer simulation, which only enables investigation of formal, knowledge-free gaming strategies. However, it makes sense to assume a *tendency* among human test takers to select ‘0’ on items for which they lack the knowledge, or do not feel confident, because this appears to be the least risky option, which will fairly often yield some points. Participants with relatively little knowledge can exploit this ‘strategy’ by answering only the few questions they are confident they know, selecting the ‘0’ on all other questions. This way, they might succeed in elevating their score to well above the −0.7 SD level. Thus, the authors’ recommendation to restrict the number of SCT items for which ‘0’ is the correct answer seems to be defensible, even if no human test taker will ever consistently choose the ‘0’ on all SCT items.

Yet, I wonder whether this recommendation will be effective. In any case, it may be hard to maintain the measure in the long run, for if an SCT is repeatedly administered, students will probably become aware that ‘0’ is rarely the most profitable option. Thus, restricting the frequency of ‘0’ as the highest yielding option in the SCT might encourage students to use the opposite gaming strategy, i.e., avoiding ‘0’. This might aggravate the problem that, viewed from probability theory, ‘0’ is an unattractive option: in practice it will be very hard to prove that a particular piece of new information does not affect the likelihood of a diagnostic hypothesis *at all*. Test takers who attempt to apply their knowledge of probability theory will realize that new information that appears to have no bearings on the current diagnostic hypothesis might nonetheless affect the likelihood of an *alternative* diagnostic hypothesis, which implicitly increases or decreases the likelihood of the *current* hypothesis (assuming they exclude each other): probabilities necessarily add up to 1. Failure to relate new information to other than the focal diagnostic hypothesis is known as ‘pseudodiagnosticity’ [3–5] and SCT, in its current form, appears to encourage this form of flawed reasoning. In contrast, in a study we observed that many students and experts are aware that alternative diagnostic hypotheses should be taken into account, because this is the way clinicians ‘think’ [6]. The point here is that apart from the gaming tendency suggested by Lubarsky and colleagues [1], there may be more reasons for test takers to prefer or avoid the ‘0’ option. If these reasons violate SCT purposes, they may compromise SCT validity.

Yet, I do not believe that gaming, or problems with the ‘0’ option, are the most pressing threats to the validity of the SCT. On the positive side, I appreciate that SCT questions are designed to avoid having single ‘correct’ or ‘consensus’ answers. Thus, in contrast to most conventional assessment tools, the SCT employs a scoring system that acknowledges an important reality in clinical practice: that even experienced clinicians often interpret data, make judgments, and respond to uncertain clinical situations in ways that vary within an acceptable range of medical practice [2, P. 185]. Or, in my words: The criterion to judge clinical reasoning is defensibility of a diagnostic hypothesis or proposed treatment, rather than correctness in a fixed normative sense. Not only in clinical practice, but in an educational context as well, exchanging arguments is part and parcel of clinical reasoning. However, not all arguments or lines of reasoning are equally defensible. Thus, when assessing students’ clinical reasoning, some standard has to be developed against which to judge this reasoning. The standard chosen by SCT designers is that the answer endorsed by the greatest proportion of expert panel members (i.e., the modal answer) yields test takers the maximum number of credit points. In fact, this seems to me a mitigated version of the classical *ad populum* (appeal to the number) fallacy: True is what many, or most, people believe. The problem is reinforced here: as SCT does not require experts to defend—or even explain—their answers, test takers who side with a minority will never know why they were denied part of the credits. This brings us directly to another problem: How can SCT test takers learn from feedback on their performance? *Can* they learn anything at all? Even if clear quantitative values could be assigned to expressions, such as ‘more likely’ and ‘almost certain,’ it is unlikely consensus can be reached on exact cutoff values. But in SCT practice, the above terms are used in a rather sloppy and intuitive way; test takers will have few, if any, clues on how to apply probabilistic reasoning. Thus, discriminating between two adjacent options will often be a matter of the respondent using his or her gut feelings, and it is hard to see any role for clinical reasoning in this. In this respect, the suggestion by Lubarsky and colleagues in their paper ‘Examining the effects of gaming and guessing on script concordance test scores’ to investigate SCT response tendencies in greater detail by asking examinees to justify their responses on SCT items [1] seems hard to apply in practice. For example, how would one judge responses, such as, “I estimate the likelihood of the diagnosis after learning the new information to be 10%; thus, I do not think it can be ruled out,” if the majority of the expert panel does believe it can be ruled out? Reasoning about likelihood or likelihood changes can never provide definitive answers, because likelihoods are determined, in the end, not by reasoning, but by collecting and analyzing empirical data.

Moreover, in my view, there is also an ethical issue here: can one justify denying students credits for answers that are endorsed by a minority of experts? Maybe if these experts’ answers are demonstrably incorrect, but this is exactly what the design of the SCT precludes. I have serious concerns about assessing students on problems or questions that show lack of agreement among experts, particularly in summative exams. Judging answers as ‘worthy of partial credit’ may sound encouraging, but in a high-stakes context, students may experience it as partial punishment rather than partial credit.

Does this mean SCT should not be used at all? Not for summative purposes, I would say. In a formative setting, on the other hand, SCT may be used to test an aspect of clinical intuition: How well do students’ responses match those of an expert panel? Clinical teachers might consider to use the test in the setting of a contest or competition, to select the student “whose thinking most closely resembles that of an experienced clinician” in a particular class. In this way, SCT can contribute, together with other small-scale educational projects, to enliven the curriculum, to add an element of entertainment and to encourage students to more intensely pursue their studies.
